# Exergy Analysis of Advanced Adsorption Cooling Cycles

**DOI:** 10.3390/e22101082

**Published:** 2020-09-26

**Authors:** Ngoc Vi Cao, Xuan Quang Duong, Woo Su Lee, Moon Yong Park, Seung Soo Lee, Jae Dong Chung

**Affiliations:** Department of Mechanical Engineering, Sejong University, Seoul 05006, Korea; caongocvi@gmail.com (N.V.C.); duongquang.mt@gmail.com (X.Q.D.); kanzest@gmail.com (W.S.L.); cavalier94@naver.com (M.Y.P.); tmdtn062@gmail.com (S.S.L.)

**Keywords:** adsorption chiller, mass recovery, heat recovery, exergy efficiency, exergy loss

## Abstract

This study conducted an exergy analysis of advanced adsorption cooling cycles. The possible exergy losses were divided into internal losses and external losses, and the exergy losses of each process in three advanced cycles: a mass recovery cycle, heat recovery cycle and combined heat and mass recovery cycle were calculated. A transient two-dimensional numerical model was used to solve the heat and mass transfer kinetics. The exergy destruction of each component and process in a finned tube type, silica gel/water working paired-adsorption chiller was estimated. The results showed that external loss was significantly reduced at the expense of internal loss. The mass recovery cycle reduced the total loss to 60.95 kJ/kg, which is −2.76% lower than the basic cycle. In the heat recovery cycle, exergy efficiency was significantly enhanced to 23.20%. The optimum value was 0.1248 at a heat recovery time of 60 s. The combined heat and mass recovery cycle resulted in an 11.30% enhancement in exergy efficiency, compared to the heat recovery cycle. The enhancement was much clearer when compared to the basic cycle, with 37.12%. The observed dependency on heat recovery time and heating temperature was similar to that observed for individual mass recovery and heat recovery cycles.

## 1. Introduction

Adsorption cooling systems (ACS) are considered a promising solution for the shortcomings of vapor compression refrigerators. ACS can be driven by low-grade heat sources (60–90 °C) such as solar energy, industrial waste heat, or exhaust heat from engines. They can also utilize eco-friendly refrigerants like water, methanol or ethanol. However, widespread application of ACS has been limited by its currently low thermal performance and bulky size.

In basic operating mode, the system suffers from a low coefficient of performance (COP) and low specific cooling power (SCP). To enhance performance, a number of advanced operating cycles have been developed, including a heat recovery cycle [1,2,3], a mass recovery cycle [4,5,6,7], and a combined heat and mass recovery cycle [8,9,10].

In our previous studies [11,12], the relative contribution of each recovery cycle for a range of heat source temperatures and cycle times was closely examined. The results showed that the mass recovery enhanced both COP and SCP by up to 24% and 37.5% at 60 °C, respectively. The enhancements in COP and SCP were 5.0% and 16.0% at a hot water temperature of 90 °C. The mass recovery was more significant at a low heating temperature than at a high heating temperature. The heat recovery increased COP by 12.56%, but reduced SCP by 10.84% at 60 °C, while a heat source of 90 °C increased COP by 11.83% and reduced SCP by 5.96%. The heat recovery process enhanced the COP but penalized the SCP, especially at low heating temperatures and short cycle times.

The energy analyses were based on the first law of thermodynamics [3,4,6,12], but the entropy or exergy analyses were based on the second law of thermodynamics. Meunier et al. [13,14] studied the effect of thermal coupling entropy production and other internal irreversibility on the COP. They introduced the concepts of external and internal thermal coupling entropy productions; the former is the entropy production between the adsorber and the heat reservoir, and the latter is due to the heat recovery phases. Pons et al. [1] introduced an entropic mean temperatures model for the heat recovery and thermal regeneration adsorption cycles with internal mass recovery. The entropic diagram was developed to examine the thermodynamic differences in the two cycle types. However, these approaches did not give any quantitative or qualitative assessment of the exergy losses occurring in the various components of the system [15].

An exergy analysis supplies the details of energy degradation for each process and component. The primary aim of an exergy analysis is to provide the causes and true magnitudes of exergy losses [16]. Until now, exergy analyses have mainly been conducted to investigate the basic adsorption cooling cycle [17,18,19,20], and rarely for advanced cycles. Moreover, the existing exergy analyses of advanced cycles have been based on the rough lumped model. Baker [21] introduced an energy and exergy model to evaluate the theoretical limit of the ideal thermal regeneration adsorption cycle. The performance of zeolite-water and silica gel-water pairs was examined over a range of maximum bed temperatures. The zeolite-water system showed a larger potential to enhance COP, by implementing thermal regeneration.

The heat recovery and mass recovery cycles are considered to be effective and simple methods for improving the thermal performance of ACS. However, there has been no study on the exergy analysis of the advanced cycles, especially based on a rigorous analysis of the sorption bed. Note that our recent research on the basic cycle showed that the rigorous results of the CFD model in sorption beds were different from those of the conventional lumped model, by approximately 15.95%, 73.8%, and 16.33% of the COP, SCP and exergy efficiency, respectively [20].

This study aims to investigate the cycle performance with mass recovery, heat recovery and the combined heat and mass recovery cycles, by exergy analysis, and compare them with those of the basic cycle to further illustrate their advantages and disadvantages. The sorption beds were rigorously modeled and the effects of heating temperature and heat recovery times were also examined.

## 2. Numerical Analysis

**Figure 1:**
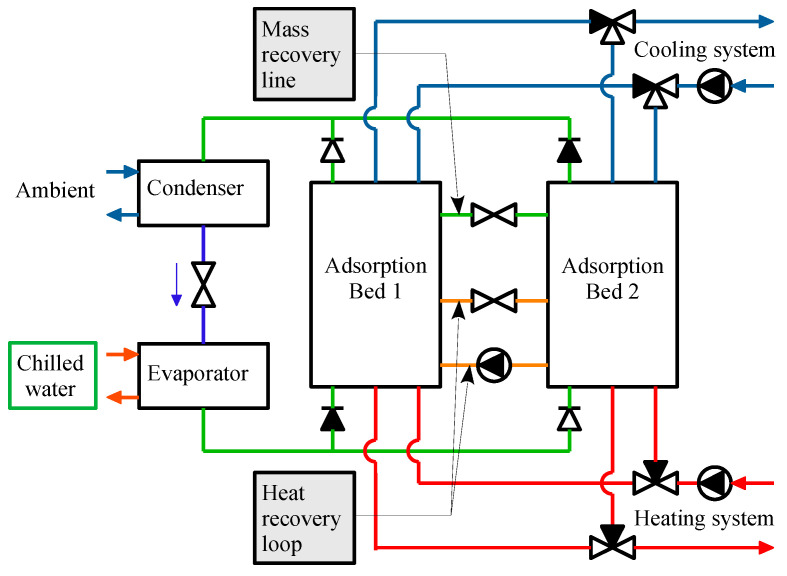
Schematic diagram of a two-bed adsorption chiller.

**Figure 2:**
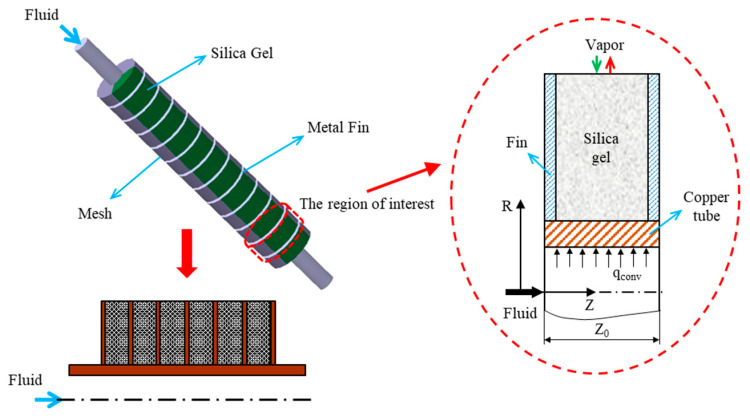
Schematic diagram of the transient two-dimensional numerical model used in the present study.

For a continuous cooling supply, multiple beds, usually in the form of two-bed systems, are commonly employed in ACS. The system consists of two adsorbers, one condenser, one evaporator, and connecting valves, as shown in Figure 1. Figure 2 shows the schematics of a transient two-dimensional numerical model used to solve the heat and mass transfer kinetics of a finned tube type, silica gel/water working paired-adsorption chiller. The parameter values and typical operating conditions are shown in Table 1. The details of the assumptions, governing equations, boundary conditions, numerical approach and their validation are available in our previous work [12,20,22]. Thus, this study shortly describes the necessary hypotheses and governing equations for the mathematical model.

### 2.1. Assumptions

The mathematical modeling was established based on the following assumptions:

- The adsorbent particles are all spherical with uniform size and porosity.
- The refrigerant vapor is an ideal gas while the adsorbed phase is a liquid.
- The thermo-physical properties of materials are constant except for the density of the refrigerant gas.
- The adsorbed and vapor phases are in local thermal equilibrium.
- The flow in the adsorption bed is axisymmetric.
- There is no heat loss through the chamber wall to the environment.
- The condenser and evaporator are idealised with a constant pressure and temperature during all processes.
- The thermochemical, kinematic and potential exergy are neglected.
- Neglecting the heat losses in the ducts and valves.
- The refrigerant is not to be sub-cooled in the condenser and not to be super-heated in the evaporator.
- The exergy destroyed in the metallic parts of the condenser/evaporator and in the secondary heat transfer fluids are negligible.
- The process in the expansion valve is considered to be isenthalpic.

### 2.2. Energy Analysis

The adsorption equilibrium for Type-RD silica gel/water working pair can be expressed by Equations (1)–(3):

(1) $$q^{*}=A\left(T_{s}\right){\left(\frac{P}{P_{0}}\right)}^{B\left(T_{s}\right)}$$

(2) $$A\left(T_{s}\right)=A_{0}+A_{1}T_{s}+A_{2}T_{s}^{2}+A_{3}T_{s}^{3}$$

(3) $$B\left(T_{s}\right)=B_{0}+B_{1}T_{s}+B_{2}T_{s}^{2}+B_{3}T_{s}^{3}$$

The numerical values of *A*_0_–*A*_3_ and *B*_0_–*B*_3_ were obtained from the experimental data reported by Khan et al. [23].

Energy balance for the adsorption bed, finned tube and heat transfer fluid are shown in Equations (4)–(6):

(4) $$\rho c_{p}\frac{\partial T_{s}}{\partial t}+\nabla \cdot \left(\rho_{v}c_{pv}{\vec{u}}_{v}T_{s}\right)=\nabla \cdot \left(k_{s}\nabla T_{s}\right)+\rho_{s}\Delta H\frac{\partial \bar{q}}{\partial t}$$

(5) $$\rho_{m}c_{pm}\frac{\partial T_{m}}{\partial t}=\nabla \cdot \left(k_{m}\nabla T_{m}\right)$$

(6) $$\rho_{f}c_{pf}\frac{\partial T_{f}}{\partial t}+\frac{\partial }{\partial Z}\left(\rho_{f}c_{p,f}u_{f}T_{f}\right)=\frac{\partial }{\partial Z}\left(k_{f}\frac{\partial T_{f}}{\partial Z}\right)+\frac{4}{D_{i}}h\left(T_{f,R=R_{i}}-T_{f}\right)$$

### 2.3. Exergy Analysis

Exergy analysis is to determine the amount of exergy destroyed in each component and process, the total exergy destroyed and the exergetic efficiency of the system. Meunier et al. [13] introduced the concepts of external and internal thermal coupling entropy production. Three sources of internal entropy production were identified: (i) desuperheating of the desorbed vapor from the adsorption equilibrium temperature *T_bed_* to the condenser temperature *T_con_*; (ii) throttling of the compensated water from the condenser pressure *P_con_* to the evaporator pressure *P_eva_*; and (iii) superheating of the vapor from the evaporator temperature *T_eva_* to the *T_bed_*. Similarly, in the present work, we used the external loss *E_D,ext_*, defined as the exergy loss due to the thermal coupling between adsorbers and cooling/heating water, and the internal loss *E_D,int_*, defined as the exergy loss during the heat and/or mass recovery phase. The other exergy losses *E_D,other_* occur in the condenser, expansion valve and evaporator.

The exergy balance for the desorption bed can be expressed by Equations (7)–(11):

(7) $${\dot{E}}_{D,bed}=\sum \left(1-\frac{T_{0}}{T_{H}}\right){\dot{Q}}_{bed}+\underset{in}{\sum }\dot{m}\psi -\underset{out}{\sum }\dot{m}\psi -\frac{d}{dt}\left(U-T_{0}S\right)$$

(8) $${\dot{Q}}_{bed}=\int_{V_{m}}\rho_{m}c_{pm}\frac{\partial T_{m}}{\partial t}dV_{m}+\int_{V_{s}}\rho_{s}\left(c_{ps}+qc_{pa}\right)\frac{\partial T_{s}}{\partial t}dV_{s}+\int_{V_{s}}\rho_{s}\frac{\partial q}{\partial t}h_{v}\left(T_{s}\right)dV_{s}+\int_{V_{s}}\rho_{s}\Delta H\frac{\partial q}{\partial t}dV_{s}$$

(9) $$\;\underset{in}{\sum }\dot{m}\psi -\underset{out}{\sum }\dot{m}\psi =\int_{V_{s}}\rho_{s}\frac{\partial q}{\partial t}\left(h_{v}\left(T_{s}\right)-T_{0}s_{v}\left(T_{s}\right)\right)dV_{s}$$

(10) $$\frac{dU}{dt}=\int_{V_{m}}\rho_{m}c_{pm}\frac{\partial T_{m}}{\partial t}dV_{m}+\int_{V_{s}}\rho_{s}\left(c_{ps}+qc_{pa}\right)\frac{\partial T_{s}}{\partial t}dV_{s}+\int_{V_{s}}\rho_{s}\frac{\partial q}{\partial t}u_{v}\left(T_{s}\right)dV_{s}+\int_{V_{s}}\rho_{s}\Delta H\frac{\partial q}{\partial t}dV_{s}$$

(11) $$\;\frac{dS}{dt}=\int_{V_{m}}\rho_{m}c_{pm}\frac{\partial \ln\left(T_{m}\right)}{\partial t}dV_{m}+\int_{V_{s}}\rho_{s}\left(c_{ps}+qc_{pa}\right)\frac{\partial \ln\left(T_{s}\right)}{\partial t}dV_{s}+\int_{V_{s}}\rho_{s}\frac{\partial q}{\partial t}s_{v}\left(T_{s}\right)dV_{s}+\int_{V_{s}}\rho_{s}\frac{\Delta H}{T_{s}}\frac{\partial q}{\partial t}dV_{s}$$

The exergy balance for the adsorption bed can be expressed by Equations (12)–(16):

(12) $${\dot{E}}_{D,bed}=\sum \left(1-\frac{T_{0}}{T_{L}}\right){\dot{Q}}_{bed}+\underset{in}{\sum }\dot{m}\psi -\underset{out}{\sum }\dot{m}\psi -\frac{d}{dt}\left(U-T_{0}S\right)$$

(13) $$\begin{array}{ll} {\dot{Q}}_{bed}= & \int_{V_{m}}\rho_{m}c_{pm}\frac{\partial T_{m}}{\partial t}dV_{m}+\int_{V_{s}}\rho_{s}\left(c_{ps}+qc_{pa}\right)\frac{\partial T_{s}}{\partial t}dV_{s}\\ & +\int_{V_{s}}\rho_{s}\frac{\partial q}{\partial t}h_{v}\left(T_{s}\right)dV_{s}+\int_{V_{s}}\rho_{s}\left(\Delta H+c_{pv}\left[T_{s}-T_{eva}\right]\right)\frac{\partial q}{\partial t}dV_{s} \end{array}$$

(14) $$\;\underset{in}{\sum }\dot{m}\psi -\underset{out}{\sum }\dot{m}\psi =\int_{V_{s}}\rho_{s}\frac{\partial q}{\partial t}\left(h_{v}\left(T_{eva}\right)-T_{0}s_{v}\left(T_{eva}\right)\right)dV_{s}$$

(15) $$\begin{array}{ll} \frac{dU}{dt}= & \int_{V_{m}}\rho_{m}c_{pm}\frac{\partial T_{m}}{\partial t}dV_{m}+\int_{V_{s}}\rho_{s}\left(c_{ps}+qc_{pa}\right)\frac{\partial T_{s}}{\partial t}dV_{s}\\ & +\int_{V_{s}}\rho_{s}\frac{\partial q}{\partial t}u_{v}\left(T_{s}\right)dV_{s}+\int_{V_{s}}\rho_{s}\left(\Delta H+c_{pv}\left[T_{s}-T_{eva}\right]\right)\frac{\partial q}{\partial t}dV_{s} \end{array}$$

(16) $$\begin{array}{ll} \;\frac{dS}{dt}= & \int_{V_{m}}\rho_{m}c_{m}\frac{\partial \ln T_{m}}{\partial t}dV_{m}+\int_{V_{s}}\rho_{s}\left(c_{ps}+qc_{pa}\right)\frac{\partial \ln T_{s}}{\partial t}dV_{s}\\ & +\int_{V_{s}}\rho_{s}\frac{\partial q}{\partial t}s_{v}\left(T_{s}\right)dV_{s}+\int_{V_{s}}\rho_{s}\left[\frac{\Delta H}{T_{s}}+c_{pv}\ln\left(\frac{T_{s}}{T_{eva}}\right)\right]\frac{\partial q}{\partial t}dV_{s} \end{array}$$

The exergy balance for the condenser can be expressed by Equations (17)–(19):

(17) $${\dot{E}}_{D,con}=\sum \left(1-\frac{T_{0}}{T_{con}}\right){\dot{Q}}_{con}+\underset{in}{\sum }\dot{m}\psi -\underset{out}{\sum }\dot{m}\psi$$

(18) $${\dot{Q}}_{con}=\int_{V_{s}}\rho_{s}\frac{\partial q}{\partial t}\left(c_{pv}\left[T_{s}-T_{con}\right]+L\left(T_{con}\right)\right)dV_{s}$$

(19) $$\underset{in}{\sum }\dot{m}\psi -\underset{out}{\sum }\dot{m}\psi =\int_{V_{s}}\rho_{s}\frac{\partial q}{\partial t}\left(\left[h_{v}\left(T_{s}\right)-h_{l}\left(T_{con}\right)\right]-T_{0}\left[s_{v}\left(T_{s}\right)-s_{l}\left(T_{con}\right)\right]\right)dV_{s}$$

The exergy balance for the evaporator can be expressed by Equations (20)–(22):

(20) $${\dot{E}}_{D,eva}=\sum \left(1-\frac{T_{0}}{T_{eva}}\right){\dot{Q}}_{eva}+\underset{in}{\sum }\dot{m}\psi -\underset{out}{\sum }\dot{m}\psi$$

(21) $${\dot{Q}}_{eva}=\int_{V_{s}}\rho_{s}\frac{\partial q}{\partial t}\left(c_{pl}\left[T_{eva}-T_{con}\right]+L\left(T_{eva}\right)\right)dV_{s}$$

(22) $$\underset{in}{\sum }\dot{m}\psi -\underset{out}{\sum }\dot{m}\psi =\int_{V_{s}}\rho_{s}\frac{\partial q}{\partial t}\left(\left[h_{l}\left(T_{con}\right)-h_{v}\left(T_{eva}\right)\right]-T_{0}\left[s_{l}\left(T_{eva}\right)-s_{v}\left(T_{eva}\right)\right]\right)dV_{s}$$

The exergy balance for the expansion valve can be obtained by Equation (23):

(23) $${\dot{E}}_{D,val}=\underset{in}{\sum }\dot{m}\psi -\underset{out}{\sum }\dot{m}\psi =\int_{V_{s}}\left(\rho_{s}\frac{\partial q}{\partial t}T_{0}\left[s_{l}\left(T_{con}\right)-s_{l}(T_{eva})\right]\right)dV_{s}$$

The exergy balance for the overall system can be expressed by Equation (24):

(24) $$E_{D}=E_{D,bed}+E_{D,con}+E_{D,val}+E_{D,eva}$$

where *E_D,bed_* is the summation of exergy destruction in the adsorption bed

(25) $$E_{D,bed}=E_{D,mrc}+E_{D,hrc}+E_{D,preh}+E_{D,heat}+E_{D,prec}+E_{D,cool}=E_{D,int}+E_{D,ext}$$

*E_D,int_* in Equation (25) denotes the exergy loss during the heat and/or mass recovery phases

(26) $$E_{D,int}=E_{D,mrc}+E_{D,hrc}$$

and *E_D,ext_* denotes the exergy loss during the heating the cooling phases by external heat sources

(27) $$E_{D,ext}=E_{D,preh}+E_{D,heat}+E_{D,prec}+E_{D,cool}$$

The input exergy supplied by heating water can be obtained using Equation (28):

(28) $$E_{in}=E\left(Q_{in}\right)=E\left(Q_{preh}\right)+E\left(Q_{heat}\right)$$

### 2.4. Performance Indices

The cycle exergetic efficiency can be expressed as Equation (29) [17]:

(29) $$\eta_{e}=1-\frac{E_{D}}{E_{in}}$$

## 3. Results and Discussion

Firstly, an energy analysis with the governing equations subject to the given boundary conditions was conducted using STAR-CCM + v9.04, a commercial CFD program, and additional user supplied codes. The grid and time step dependence were thoroughly tested. The computation time was approximately 24 h for a typical model running on an Intel^®^ Xeon^®^ CPU E3-1275 v3 @3.50GHz.

Secondly, based on the information obtained from the energy analysis, an exergy analysis was performed using MATLAB code to estimate the exergy destruction in each process and component, and then the exergy efficiency of the system was evaluated.

### 3.1. Effect of Recovery Strategy

**Figure 3:**
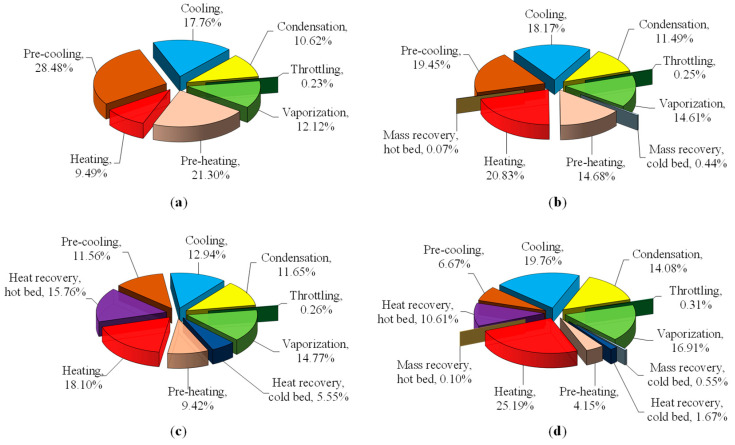
Exergy loss in each process of the different adsorption cooling cycles: (**a**) Basic cycle. (**b**) Mass recovery cycle. (**c**) Heat recovery cycle. (**d**) Combined heat and mass recovery cycle.

The results of the exergy analysis for the typical working conditions in Table 1 are given in Figure 3 and Table 2. Exergy losses in each process for the different adsorption cooling cycles including the basic cycle, mass recovery cycle, heat recovery cycle, and the combined heat and mass recovery cycle, were compared.

#### 3.1.1. Basic Cycle

Figure 3 shows that all of the exergy losses in the basic cycle belong to the external losses because the adsorber is totally heated up from the coldest state (or cooled down from the hottest state) by heating (or cooling) water supplied to the system. Due to the temperature difference between the adsorber and external heat source, the exergy loss of the adsorber is the largest accounting for nearly 77% of the total exergy losses of the system. Therefore, the exergy efficiency of the basic cycle is the lowest, at 0.1013, compared to the other advanced cycles. This is in good agreement with our previous findings [20].

#### 3.1.2. Mass Recovery Cycle

During the mass recovery cycle, the desorbed hot vapor moves directly from the hot bed to the cold bed. It smooths out the temperature difference between the heat transfer fluid (HTF) and the adsorber, and leads to shorter pre-heating and pre-cooling processes, and a larger amount of circulating refrigerant. Thus, smaller external loss is expected.

Usually the mass recovery time is very short, here 4s; thus, the amount of the internal loss, 0.31 kJ/kg, is very small compared to the external loss of 44.57 kJ/kg. The numerical results show that the mass recovery cycle reduces the external loss to 44.57 kJ/kg, but increases *e_D,other_* to 16.06 kJ/kg, which corresponds to −7.68% and +11.53%, respectively, compared to the basic cycle.

Overall, the total loss decreases to 60.95 kJ/kg (−2.76%). The reason is that the mass recovery results in a larger amount of water vapor to be desorbed/adsorbed during the desorption/adsorption process. Therefore, the useful output exergy, *e_eva_*, is enhanced by 18% (from 7.06 kJ/kg to 8.33 kJ/kg), and the exergy efficiency, *η_e_*, is improved to 0.1203.

The internal exergy losses in the hot and cold adsorbers are different. Because the internal loss equals the exergy of the outflow vapor, the internal loss in the hot adsorber is relatively small (9.2 × 10^−3^ kJ/kg or 0.07% of total loss). On the other hand, the internal loss in the cold adsorber comes from the inflow exergy, and is much higher (58.5 × 10^−3^ kJ/kg or 0.44% of total loss) than that of the hot adsorber. This observation is in accord with [1].

#### 3.1.3. Heat Recovery Cycle

During the heat recovery phase, the external loss (*e_D,ext_* = 24.09 kJ/kg) is reduced to half of the basic cycle (48.28 kJ/kg). The internal loss, *e_D,int_* = 9.87 kJ/kg, is about half of the external loss, and the total loss, *e_D_* = 46.31 kJ/kg, equals one-third of the basic cycle. As a consequence, the *η_e_* is significantly enhanced, to 23.20%. Note that the internal loss in the heat recovery cycle relates to the amount of recovered heat, thus it is much higher than that of the mass recovery cycle.

The internal loss in the hot adsorber (15.76%) during the heat recovery phase is about three times higher than in the cold adsorber (5.55%). This is because the drop in temperature in the hot adsorber is larger than the increase in temperature in the cold adsorber in the basic cycle for the same operating conditions of pressure and temperature. The internal loss during the heat recovery phase significantly reduces the external exergy loss in the pre-heating (21.30% → 9.42%) and pre-cooling (28.48% → 11.56%) phases, because the pre-heating/cooling time is shortened by introducing the heat recovery.

#### 3.1.4. Combined Heat and Mass Recovery Cycle

The combined heat and mass recovery cycle exploits the merits of both former advanced cycles. The exergy losses are shown in Table 2, compared to the basic cycle and the former advanced cycles. The external loss (26.88 kJ/kg) and the other exergy losses (15.09 kJ/kg) are slightly higher than those of the heat recovery cycle, but the internal loss (6.23 kJ/kg) decreased to two-thirds that of the heat recovery cycle. Thus, the overall total loss (48.19 kJ/kg) is slightly higher than the heat recovery cycle. Because the useful exergy is remarkably improved (17.8%, from 6.60 to 7.77 kJ/kg), the *η_e_* = 0.1389 is enhanced up to 11.30% compared to the heat recovery cycle (*η_e_* = 0.1248). The enhancement is much clearer when compared to the basic cycle (*η_e_* = 0.1013) with 37.12%.

As in the mass recovery cycle, the internal loss caused by the mass recovery phase in the cold adsorber (0.55% of total loss) is almost five times higher than that of the hot adsorber (0.1%). In contrast, the internal loss due to the heat recovery phase in the hot adsorber (10.61%) is about six times that of the cold adsorber. The external loss during the pre-heating/cooling periods decreased considerably, thanks to the very short duration of these phases, with 4.15% and 6.67% of exergy loss for the pre-heating phase and the pre-cooling phase, respectively. The heating/cooling processes are the dominant processes in the external loss, accounting for 25.19% and 19.76%, respectively. Because the mass recovery results in a larger amount of cyclic water vapor, the other internal exergy losses including condensation, throttling and evaporation were also increased.

### 3.2. Effect of Heating Temperature

#### 3.2.1. Mass Recovery Cycle

**Figure 4:**
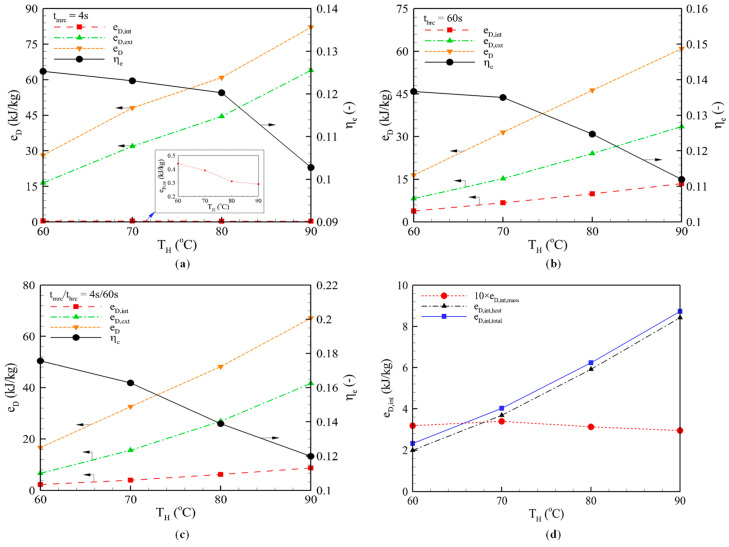
Effect of heating temperature on the performance of: (**a**) Mass recovery cycle. (**b**) Heat recovery cycle. (**c**,**d**) Combined heat and mass recovery cycle.

Figure 4a shows the exergy losses and exergy performance of the mass recovery cycle at different heating temperatures. The dependency on the heating temperature appears proportional to the external loss, but reversely proportional to the internal loss due to the reduction in the amount of water vapor. Because the desorption process is faster at high heating temperatures, the amount of water vapor desorbed/adsorbed during the mass recovery phase decreases as the heating temperature increases. Higher internal loss and lower external loss are expected for the larger amount of recovered vapor. Because the major exergy loss comes from the external loss, the total exergy loss increases, and thus *η_e_* deceases, as the temperature increases. That is the reason why the mass recovery is less influential at low heating temperature, as reported in our previous work [12].

#### 3.2.2. Heat Recovery Cycle

The influence of the heating temperature on the exergy losses and performance of the heat recovery cycle at a fixed heat recovery time of *t_hrc_* = 60 is examined in Figure 4b. Both the internal and external exergy losses increased as the heating temperature increased. For instance, the internal, external and total exergy losses increased by almost four times when the heating temperature increased from 60 °C to 90 °C.

The increase in internal loss comes from the larger amount of heat recovered at the higher heating temperature. Meanwhile, the increase in external loss comes from the greater cycling mass of refrigerant vapor. At a high heating temperature, the amount of water vapor, which would be desorbed during the desorption process and then be re-adsorbed during the adsorption process, is larger than that at a low heating temperature, resulting in the bigger mass of refrigerant circulation in a cycle. The greater the cycling mass of refrigerant, the more input heat energy needs to be supplied. Accordingly, the external loss increases and the total loss climbs, which contributes to a decrease in *η_e_*.

#### 3.2.3. Combined Heat and Mass Recovery Cycle

Figure 4c shows the system performance at different heating temperatures. The mass recovery time and heat recovery time were fixed at 4 s and 60 s. The internal exergy losses of the combined heat and mass recovery cycle are the summation of the internal loss from mass recovery and the loss from the heat recovery phase. The internal loss of the mass recovery phase decreased with the higher heating temperature, while the internal loss of the heat recovery phase showed the opposite direction. The reason was explained in Section 3.2.1 and Section 3.2.2. However, the magnitude of the internal loss in the mass recovery phase was much smaller than that in the heat recovery phase. Consequently, the total internal loss increased with increasing heating temperature, as shown in Figure 4d. For example, the internal loss decreased from *e_D,int_* = 0.32 kJ/kg to *e_D,int_* = 0.3 kJ/kg, whereas the external loss increased from 2 kJ/kg at *T_H_* = 60 °C to 8.43 kJ/kg at *T_H_* = 90 °C, respectively. Thus, the *η_e_* sharply decreased by 31.71%.

### 3.3. Effect of Heat Recovery Time

#### 3.3.1. Heat Recovery Cycle

**Figure 5:**
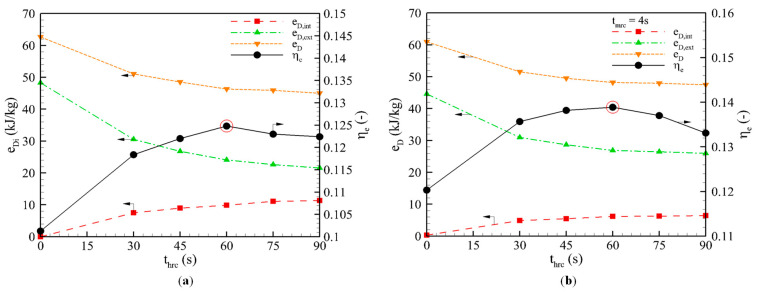
Influence of heat recovery time on the exergy losses and performance of the: (**a**) Heat recovery cycle. (**b**) Combined heat and mass recovery cycle.

Figure 5a shows the variation in the exergy loss and *η_e_* along with heat recovery time. As the recovery time increases, the internal loss increases, which leads to a reduction in the external loss and the total loss. The external loss is reduced to a half after around *t_hrc_* = 60 s. Note that the extension of the heat recovery time shortens the effective adsorption/desorption periods, which results in a decrease in useful exergy. Thus, the *η_e_* increases to a peak of 0.1248 at *t_hrc_* = 60 s, and then decreases as the heat recovery time increases. This observation on optimum heat recovery time is consistent with our earlier results based on energy analysis [12].

#### 3.3.2. Combined Heat and Mass Recovery Cycle

Figure 5b shows the exergy losses and performance of the combined heat and mass recovery cycle at different heat recovery times. Due to the short mass recovery time, the internal loss is mainly caused by the heat recovery phase. The longer the heat recovery time, the larger the amount of recovered heat and the higher the observed internal loss is. However, the external loss and the total loss decrease as the recovery time increases. Similar to the heat recovery cycle, the *η_e_* reached an optimal value at *t_hrc_* = 60 s.

## 4. Conclusions

This study investigated cycle performance with mass recovery, heat recovery and combined heat and mass recovery cycles using exergy analysis. The well-established numerical analysis of a finned-tube type adsorption chiller was extended to an exergy analysis. The type, location, magnitude and relation between the possible exergy losses occurring in each type of cycle were analyzed. The sorption beds were rigorously modeled and the effect of heating temperature and heat recovery time were also closely examined. The following results were obtained.

1. Mass recovery cycle: The shorter pre-heating and pre-cooling phases, and the larger amount of circulating refrigerant, reduced the external exergy losses to 44.57 kJ/kg, but increased *e_D,other_* to 16.06 kJ/kg, which corresponds to −7.68% and +11.53%, respectively, compared to the basic cycle. Overall, the total exergy losses decreased to 60.95 kJ/kg (−2.76%). As the temperature increased, the total exergy loss increased and thus, *η_e_* deceased. This explains why the mass recovery is less influential at low heating temperatures.
2. Heat recovery cycle: The external loss (*e_D,ext_* = 24.09 kJ/kg) was reduced to half of the basic cycle (48.28 kJ/kg) and the internal loss, *e_D,int_* = 9.87 kJ/kg, was about half of the external loss. The total exergy losses, *e_D_* = 46.31 kJ/kg, were equal to one-third of the basic cycle. As a consequence, the *η_e_* was significantly enhanced, to 23.20%. The *η_e_* showed an optimum value of 0.1248 at *t_hrc_* = 60 s, and both the internal and external exergy losses increased as the heating temperature increased.
3. Combined heat and mass recovery cycle: The increase in external exergy losses was compensated by the decrease in internal exergy losses, and thus, the total loss was slightly higher than the heat recovery cycle. However, the useful exergy had a remarkable improvement (17.8%, from 6.60 to 7.77 kJ/kg), which resulted in 11.30% enhancement of *η_e_* = 0.1389 compared to the heat recovery cycle (*η_e_* = 0.1248). The enhancement is much clearer when compared to the basic.
4. cycle (*η_e_* = 0.1013) with 37.12%. The dependency on heat recovery time and heating temperature was similar to the observation of the individual mass recovery and heat recovery cycles.

## Figures and Tables

**Table 1:** Parameter values and working conditions.

Parameter	Symbol	Values
Fin Pitch	*Z_o_*	3 mm
Fin Height	*L_o_*	10 mm
Fin Thickness	*δ*	0.4 mm
Inner Diameter of Metal Tube	*D_i_*	10 mm
Outer Diameter of Metal Tube	*D_o_*	12 mm
Particle Diameter	*d_p_*	200 μm
Evaporator Temperature	*T_eva_*	15 °C
Condenser Temperature	*T_con_*	30 °C
Cooling Temperature	*T_L_*	30 °C
Heating Temperature	*T_H_*	60 °C~90 °C
Average Fluid Velocity	*u_f_*	1 m/s
Density of Adsorbent	*ρ_b_*	761 kg/m^3^
Specific Heat of Adsorbent	*C_p.b_*	920 J/kgK
Thermal Conductivity of Adsorbent	*k_b_*	0.198 W/mK
Heat of Adsorption	Δ*H*	2.76 × 106 J/kg
Porosity of the Particle	*ε_p_*	0.43
Porosity of the Bed	*ε_b_*	0.36
Cycle Time	*t_cycle_*	840 s
Mass Recovery Time	*t_mrc_*	4 s
Heat Recovery Time	*t_hrc_*	≤ 90 s

**Table 2:** Exergy balance for different adsorption cooling cycles.

Cycle	*t_hrc_* (s)	*t_mrc_* (s)	Specific Exergy (kJ/kg)					*η_e_* (−)
			*e_D,int_*	*e_D,ext_*	*e_D,other_*	*e_D_*	*e_eva_*	
Basic Cycle	0	0	0.00	48.28	14.40	62.68	7.06	0.1013
Mass Recovery Cycle	0	4	0.31	44.57	16.06	60.95	8.33	0.1203
Heat Recovery Cycle	60	0	9.87	24.09	12.35	46.31	6.60	0.1248
Combined Heat and Mass Recovery Cycle	60	4	6.23	26.88	15.09	48.19	7.77	0.1389

## Abbreviations

**Nomenclature**	
*c_p_*	Specific heat (J/kg K)
*d_p_*	Particle diameter (μm)
*D*	Diameter (mm)
*E*	Exergy (J)
*e*	Specific exergy (kJ/kg)
Δ*H*	Heat of adsorption (J/kg)
*h*	Heat transfer coefficient (W/m^2^ K)
*k*	Thermal conductivity (W/m K)
*L_o_*	Length (mm)
*ṁ*	Mass flow rate (kg/s)
*P*	Pressure (Pa)
*Q*	Heat (J)
*q*	Water uptake (kg/kg)
*S*	Entropy (J/K)
*T*	Temperature (K)
*U*	Internal energy (J)
*V*	Volume (m^3^)
*t*	Time (s)
*u*	Velocity (m/s)
*Z_o_*	Fin pitch (mm)
**Greek symbols**	
*δ*	Fin thickness (mm)
*ε*	Porosity (-)
*η*	Exergy efficiency (-)
*ρ*	Density (kg/m^3^)
*ψ*	Mass exergy (J)
**Subscripts**	
*a*	Adsorbed vapor
*bed*	Adsorbent bed
*con*	Condenser
*cool*	Cooling process
*cycle*	Cycle
*D*	Destruction
*ext*	External
*eva*	Evaporator
*H*	Heating fluid
*heat*	Heating
*hrc*	Heat recovery
*i*	Inner tube diameter
*in*	Inlet
*int*	Internal
*L*	Cooling fluid
*l*	Liquid phase
*m*	Metallic metal
*mrc*	Mass recovery
*o*	Outer tube diameter
*other*	Other
*out*	Outlet
*prec*	Pre-cooling
*preh*	Pre-heating
*s*	Solid adsorbent
*v*	Vapor
*val*	Valve
